# Tartar

**Published:** 1868-09

**Authors:** Andrew S. Cutler


### ARTICLE III.

### *Tartar.*

By Andrew S. Cutler, D. D. S.

Tartar is that peculiar substance, which, under various pathological phenomena, is found deposited upon the surfaces of teeth, frequently collecting in considerable quantity about their necks, and insinuating its concretions farther and farther down upon their roots, often producing such a complication and intensity of diseased action, as would seem almost incredible. For instance, there may be exfoliation and absorption of the gums, causing the teeth to become loosened, and not unfrequently, drop out of themselves: there may be, also, tumorous, and spongy excrescences of the gums, a greater or less degree of hemorrhage, and occasionally such an abnormal condition of the alveolar process and various portions of the maxillary bone, as to cause necrosis and exfoliation of the parts. This disordered condition also implies a derangement of the digestive tract, impure breath, and very likely, the accompainments of cough, head ache, and diarrhœa. There may be, also, melancholy, and a general depression of the nervous system, occasionally amounting to absolute prostration. There is little doubt but that this deleterious concretion is a deposit chiefly from saliva, with a large admixture of mucus. In fact chemical analysis often reveals a proportion of this latter substance, as we see from the table of Vauquelin and Langier—

| | |
|---|---|
| Phosphate of lime and a little Manganese...... | 66 |
| Carbonate of lime............. ...................... | 9 |
| Salivary mucus (including ptyaline).............. | 13 |
| Animal matter soluble in hydrochloric acid..... | 5 |
| Water and loss......................... ............. | 7 |

A close analysis of saliva and mucus, reveals the necessary material in sufficient quantity to form these concretions. Bidder and Schmidt make the phosphates and carbonates amount to very nearly one per cent in the saliva, and in either case the material is in ample amount to account for the concretions from the stated sources. All that is necessary is that the surfaces of the teeth should have a sufficient affinity for the substance in question, to cause a nucleous for deposit, and of this there can be no doubt. Different varieties of tartar vary widely in their nature and appearance according to the physiological, or pathological condition of the individual. In strong robust constitutions, it is seldom found in large quantities. Its color, in such cases is of a dark brown, and of a texture very dense. Where there is a decidedly billious diathesis, there is usually an increased tendency to this deposit, which under this condition of things is of a lighter shade, and more readily dissolved by the action of certain acids. White tartar is indicative of a temperament peculiarly mucous. It is dissolved by the alkalies, while the action of acid upon it is scarcely perceptible. Under such a variety of physical conditions, their chemical analysis must also widely vary. In most kinds of tartar, there is a notable superabundance of the phosphates and carbonates, while in other varieties there is nearly 40 per cent of purely animal matter. Hence the difference in action upon them by acids and alkalies. Of the animal matter entering into the composition of tartar, fibrin, animal fat and mucus are in largest proportion. M. Mandl, believed this concretion to be made up of infusoria. Scherer, also detected them in large numbers in the saliva of a girl suffering from a scorbutic affection. These however, undoubtedly had their origin in the vitiated mucus always a component of tartar and mixed with the saliva of this cavity. Berzelins makes the following analysis of tartar.

| | |
|---|---|
| Phosphate of lime and magnesia | 79. |
| Salivary mucus | 12.5 |
| Ptyaline | 1. |
| Animal matter soluble in hydrochloric acid | 7.5 |
| | 100.0 |

The following table of Mr. Pepys shows a larger per cent. of animal matter, and a corresponding less amount of mineral substance.

| | |
|---|---|
| Phosphate of lime | 35. |
| Fibrin, or cartilage | 9. |
| Animal fat, or oil | 3. |
| Loss | 3. |
| | 50. |

It will be seen from the foregoing analysis, that this specimen of tartar contained upwards of 24 per cent. of animal matter. Of course it was one of the softer varieties, and of a lighter color than the denser calculi. With regard to the use of acids for removing these unhealthy concretions, (an imposition not unfrequently perpetrated upon persons ignorant of the chemistry of these organs) nothing can be more deleterious. Yet it is frequently done upon the supposition that the acid is of that degree of dilution to possess a stronger affinity for the mineral substance of the concretion than it has for the tooth itself. No reasoning could be more fallacious; for the mineral components of the teeth, and the calcareous depositions upon them, being composed of essentially the same chemical ingredients. Any acid that would decompose the tartar, would also exert a materially hurtful action upon the teeth. As a general rule, the darker varieties of tartar indicate a naturally good constitution. Where, however, the physical energies have been greatly enervated by dissipation or disease, the accumulation of this concretion may become excessive, and, unless removed, give rise to results truly unfortunate. The lighter shades of this peculiar substance, contain, as already stated a larger proportion of animal matter, and are of a condition of friability from being easily crushed by the fingers, to a degree of consistency no denser than the curd of cheese.

